# Neuropsychiatric Atypical Manifestation in Wilson's Disease: A Case Report and Literature Review

**DOI:** 10.7759/cureus.9290

**Published:** 2020-07-20

**Authors:** Shubhashree Page, Likhita Shaik, Romil Singh, Sawai Singh Rathore, Kaushal Shah

**Affiliations:** 1 Surgery, Mahatma Gandhi Institute of Medical Sciences, Wardha, IND; 2 Internal Medicine, Mayo Clinic, Rochester, USA; 3 Internal Medicine, Metropolitan Hospital, Jaipur, IND; 4 Internal Medicine, Dr. Sampurnanand Medical College, Jodhpur, IND; 5 Psychiatry, Griffin Memorial Hospital, Norman, USA

**Keywords:** neuropsychiatry, psychosis, kayser fleischer ring, hepatomegaly, ceruloplasmin, wilson's disease, kayser-fleischer ring

## Abstract

Wilson's disease (WD) is a rare genetic disorder of copper metabolism that often manifests several clinical signs at the time of diagnosis. Typically it affects the liver in the early stages of the disease course and tends to show neuropsychiatric involvement in the later stages. Early diagnosis of WD holds a prognostic value, and an atypical presentation of the disease adds complexities in diagnosis. Even though we need to consolidate further the treatment guidelines for managing psychiatric and neurological symptoms optimally in the patients of WD, identifying signs at the early stages of the disease is crucial to avoid its detrimental effects on the human body. In this case presentation, a patient with no family history of psychiatric condition showed an early onset of neuropsychiatric symptoms without any other clinical signs of WD. Through this clinical case, we emphasize the importance of ruling out WD in patients that predominantly presents with psychiatric symptoms as a lone symptom. It also highlights the possible diagnostic value and significance of the ceruloplasmin level in identifying WD disease in early stages, when other clinical signs are absent, including liver abnormalities.

## Introduction

Wilson's disease (WD) is a rare autosomal recessive inherited disorder of a hepatolenticular degeneration, described first by Samuel Alexander Kinnier Wilson in 1912 [1]. The incidence of WD is estimated to be one in 30,000 births [2]. It is caused by a mutation in the ATPase copper-transporting beta (ATP7B) gene coding for a membrane-bound copper-transporting ATPase, leading to its absent or reduced function [3]. Per the normal human physiology, the liver produces and secretes ceruloplasmin without copper, which is known as apoceruloplasmin, and later it binds with copper to eradicate its excess amount through bile [4]. The gene mutation in WD leads to a failure of copper transport in hepatocytes and impairment of incorporation into the ceruloplasmin that decreases the excretion of copper into the bile resulting in liver tissue injury due to excess amount of copper accumulation [4,5]. In WD, a decreased blood level of the ceruloplasmin is found in the patients due to reduced half-life of apoceruloplasmin. Copper from the blood eventually gets deposited in other organs, including the brain, kidneys, and cornea. It causes a wide variety of clinical symptoms due to the toxic effect of copper that chiefly involves the liver and brain [4]. WD typically presents with hepatic manifestation in childhood and neuropsychiatric presentation in adults. At the time of diagnosis, about 60% shows liver function abnormalities, 40% to 60% exhibit neurological symptoms, and up to 10% to 25% present with psychiatric symptoms [5]. Kayser-Fleischer (K-F) ring is observed in 90% to 100% of the patients with pronounced neurological signs and 20% to 30% of asymptomatic patients. Even though the neurological and psychiatric symptoms can manifest at any stage of the disease, it usually exhibits signs in the later stages of the condition after the occurrence of the liver symptoms [5,6]. Here we illustrate an atypical case of WD presenting with neuropsychiatric signs as the primary presenting sign before exhibiting any significant symptoms of liver dysfunctions.

## Case presentation

A 23-year-old South East Asian woman with no known past psychiatric history presented with psychosis, sleep disturbances, and nightmares. On admission, the patient was conscious, disoriented, delusional, irritable, anxious, confused, and had incoherent speech. There was staccato speech and mild wing beating low-amplitude tremors observed during the examination, which was never noticed before the admission by her family members. According to her family, the patient showed paranoid behavior and loss of emotional control for the last two days. The patient's family history indicates confirmed WD in elder brother, who experienced mild right hand weakness in the past with no other symptoms. Her vital signs were within normal limits, and laboratory studies showed no abnormalities except subnormal ceruloplasmin level of 14 mg/dL. The positive family history of WD, the presence of neuropsychiatric symptoms, and a low level of ceruloplasmin directed the diagnosis towards WD. The patient was admitted to an inpatient facility and started on D-penicillamine (DPA) 250 mg by mouth (PO) three times a day (TID) with once daily 25 mg pyridoxine hydrochloride. Treatment resulted in a gradual improvement of the patient's condition within a week of medication initiation. Hence, it was concluded that the neuropsychiatric symptoms were attributed to WD.

**Figure 1:**
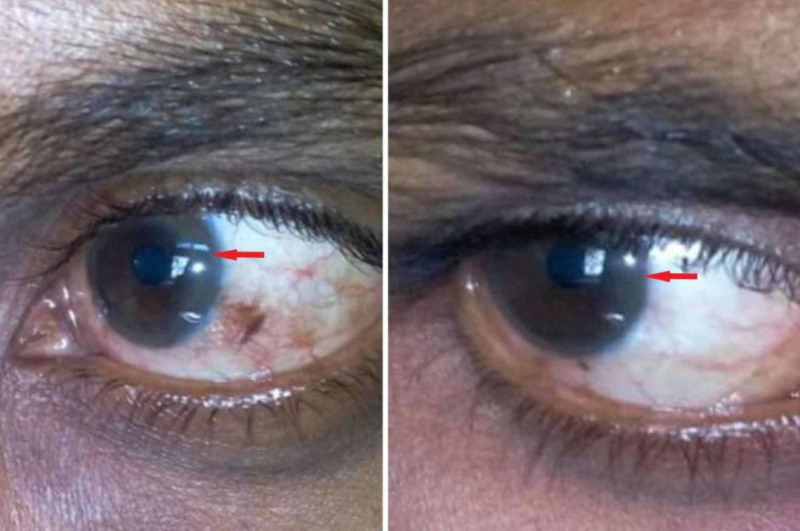
Ocular examination with a pen torch showing dense brown Kayser-Fleischer ring in both eyes

**Figure 2:**
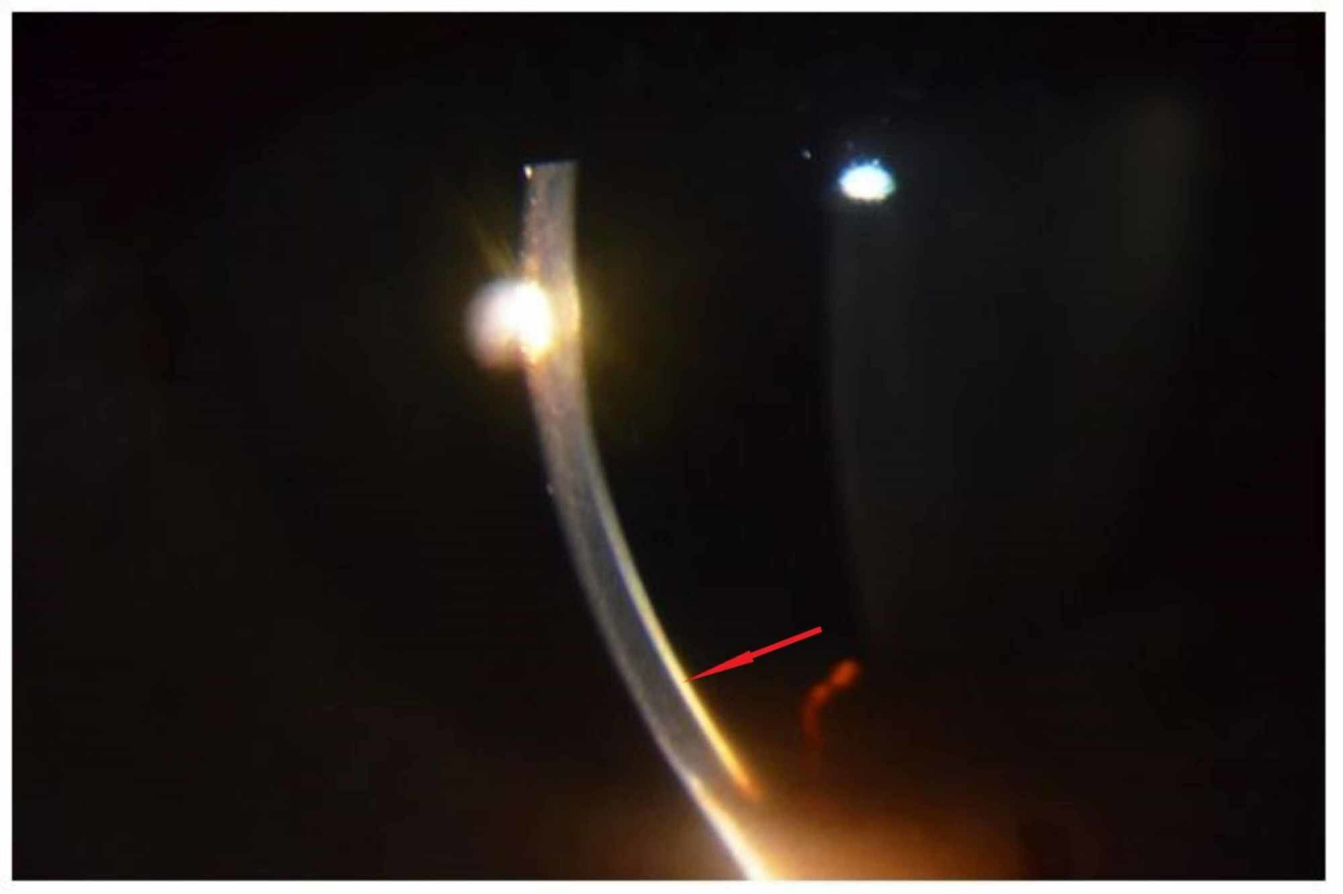
Slit illumination showing Kayser-Fleischer ring

About three months from the initial visit, the patient was readmitted because of psychosis resulting from the non-maintenance of the therapy. At this second visit, the patient also presented rhythmic bilateral prominent tremors, amplified upon the extension of both arms. Upon ocular examination, we identified the first time presence of a dense brown ring on the cornea near the limbus of both eyes. Ocular pen torch (Figure 1) and slit-lamp eye examination (Figure 2) revealed the presence of copper deposition in the cornea at the level of Descemet's membrane, confirming the presence of 'Kayser-Fleischer' ring.

Abdominal examination revealed splenomegaly of about 3 cm below the left costal margin in the midclavicular line; however, no hepatomegaly was appreciated. Laboratory findings revealed total bilirubin 0.86 mg/dL, direct bilirubin 0.55 mg/dL, indirect bilirubin 0.31 mg/dL, alanine aminotransferase/serum glutamic oxaloacetic transaminase 16 IU/L, aspartate aminotransferase/serum glutamic pyruvic transaminase 14 IU/L, alkaline phosphatase 80 IU/L, albumin 3.9 g/dL, and albumin to globulin ratio of 1.44.

The Leipzig or Ferenci scoring system to screen WD confirmed the diagnosis. It accounts for the parameters such as K-F rings, neuropsychiatric symptoms, serum ceruloplasmin levels, Coombs-negative hemolytic anemia, liver size, urine 24-hour copper excretion, and mutation analysis. The WD diagnosis is established with scores equal to or greater than 4. This patient scored five points [7]. In addition to the findings from the second visit, resuming the initially prescribed treatment regimen without using emergency antipsychotic drugs showed improvement in the patient's condition.

## Discussion

Although WD diagnosis was confirmed based on the finding at the second visit, the presenting clinical scenario from the first visit with lone neuropsychiatric symptoms and subnormal ceruloplasmin level posed a challenge in diagnosing and managing this patient.

WD is a rare, autosomal recessive disorder caused by a mutation in ATP7B coding for metal‐transporting P‐type adenosine triphosphatase (ATPase) that leads to decreased hepatocellular excretion of copper into bile causing hepatic accumulation of copper [8]. Copper first accumulates in the liver and then gets reallocated and accumulated in body's organs, including the brain, spleen, kidneys, and cornea [9]. Typically patients present first with a hepatic presentation during the first or second decade of life and later show neurological or psychiatric symptoms in the third or fourth decade of life [10]. In addition to the liver symptoms or enzymes abnormalities in WD, only about 40% to 60% exhibit neurological symptoms, and 10% to 25% present with psychiatric symptoms at the time of diagnosis. Psychiatric or neurological symptoms can occur at any stage of the disorder, but unlikely without primary liver abnormalities [5]. However, this patient experienced pronounced psychiatric and mild neurological symptoms at the beginning of WD with a subnormal level of ceruloplasmin.

In WD, psychiatric and neurological symptoms are secondary to cerebral copper deposition. The neurological signs could be involuntary movements (tremor, dystonia, ataxia, parkinsonian syndrome, chorea), speech disturbances, dysphagia, autonomic dysfunction (orthostatic hypotension, electrocardiographic abnormalities, salivation), or gait problems [5,11,12]. Psychiatric symptoms could be related to personality disorders (irritability, antisocial behavior, disinhibition), mood disorders (depression, bipolar disorders, suicidal attempts), psychosis, anorexia, sleep disturbances, and cognitive impairment [5]. Anxiety, depression, sleep disturbances, tremor, dysarthria, or gait disturbances can occur by itself or a part of neurodegenerative diseases, and the absence of a typical WD clinical scenario frequently results in delays in diagnosis and management. Timely diagnosis and management of WD play a significant prognostic role as early therapy improves symptoms without further deterioration [12,13].

The diagnosis of WD is established by considering several factors, such as family history, clinical scenario, serum ceruloplasmin level, K-F ring, urinary copper concentration, hepatic copper concentration, and liver enzymes abnormalities [5,14]. K-F rings reflect copper deposition in cornea's Deçemet's membrane, which appears near the limbus as a golden-brown pigment band. The identification of K-F rings in most patients requires a slit-lamp examination by an experienced observer. They are not pathognomonic to WD, as it is also seen in patients with chronic cholestasis. However, the WD patients with the neurological presentation almost always present with the K-F ring [8]. MRI is a sensitive technique for identifying the progression in the later stage of WD. The excess deposition of copper causes neural loss, gliosis, fiber deterioration, and vacuolation linked to increased brain water content [15]. Even though a low ceruloplasmin level is linked to renal disorder and enteric protein loss, no study per our knowledge investigated explicitly regarding its diagnostic value in patients with positive WD family history. It is crucial to study the role of ceruloplasmin in the context of neuropsychiatric symptoms when suspecting WD, as less than 5 mg/dL has shown substantial evidence for WD diagnosis [8].

Penicillamine is the primary anti-copper treatment used in WD, including for alleviating the severity of its neuropsychiatric manifestation [8]. Due to the higher toxicity profile of penicillamine, DPA is preferred orally in a dosage of 250-500 mg thrice a day [15]. As DPA interferes and antagonizes pyridoxine, a daily oral dose of pyridoxine hydrochloride 25-40 mg is recommended [16].

## Conclusions

In summary, not all patients of WD with neuropsychiatric symptoms present with coexisting clinical problems of the liver at the time of diagnosis. It is eminent to exclude WD in a patient presenting with neuropsychiatric symptoms. The association of ceruloplasmin with WD needs further research to uncover its potential diagnostic role in patients. We aim to emphasize the critical aspect of diagnosing, as neuropsychiatric symptoms without typical clinical liver abnormality involvement can be fatal if not treated appropriately. Therefore, timely diagnosis and management of WD hold prognostic value to reduce disease impact, and clinicians should be vigilant to identify similar atypical scenarios.
